# Pathogenic mtDNA mutations causing mitochondrial myopathy: The need for muscle biopsy

**DOI:** 10.1212/NXG.0000000000000082

**Published:** 2016-06-23

**Authors:** Steven A. Hardy, Emma L. Blakely, Andrew I. Purvis, Mariana C. Rocha, Syeda Ahmed, Gavin Falkous, Joanna Poulton, Michael R. Rose, Olivia O'Mahony, Niamh Bermingham, Charlotte F. Dougan, Yi Shiau Ng, Rita Horvath, Doug M. Turnbull, Grainne S. Gorman, Robert W. Taylor

**Affiliations:** From the Wellcome Trust Centre for Mitochondrial Research, Institute of Neuroscience (S.A.H., E.L.B., A.I.P., M.C.R., S.A., G.F., Y.S.N., D.M.T., G.S.G., R.W.T.), The Medical School, Institute of Genetic Medicine (R.H.), Newcastle University; Nuffield Department of Obstetrics and Gynaecology (J.P.), University of Oxford; Department of Neurology (M.R.R.), King's College Hospital NHS Foundation Trust, London; Departments of Neurology and Neuropathology (O.O., N.B.), Cork University Hospital, Ireland; and The Walton Centre for Neurology and Neurosurgery (C.F.D.), Liverpool, UK.

Pathogenic mitochondrial tRNA (mt-tRNA) gene mutations represent a prominent cause of primary mitochondrial DNA (mtDNA)-related disease despite accounting for only 5%–10% of the mitochondrial genome.^1,2^ Although some common mt-tRNA mutations, such as the m.3243A>G mutation, exist, the majority are rare and have been reported in only a small number of cases.^3^ The *MT-TP* gene, encoding mt-tRNA^Pro^, is one of the less polymorphic mt-tRNA genes, and only 5 *MT-TP* mutations have been reported as a cause of mitochondrial muscle disease to date (table e-1 at Neurology.org/ng, P6–10). We report 5 patients with myopathic phenotypes, each harboring different pathogenic mutations in the *MT-TP* gene, highlighting the importance of *MT-TP* mutations as a cause of mitochondrial muscle disease and the requirement to study clinically relevant tissue.

## Methods.

All patients were referred to our specialist mitochondrial laboratory in Newcastle upon Tyne, UK. Diagnostic muscle biopsies were analyzed by standard histopathologic and biochemical techniques and a quadruple immunofluorescent assay to assess the expression of mitochondrial complex I and complex IV subunits.^4^ DNA was extracted using standard protocols; sequencing of the entire mitochondrial genome (GenBank Accession Number NC_012920.1) was undertaken using muscle DNA. Pyrosequencing was used to quantitatively assess mtDNA mutation heteroplasmy levels in available tissues and in laser-captured individual cytochrome *c* oxidase (COX)-positive and COX-deficient fibers.^2^

## Results.

**Figure 1.**
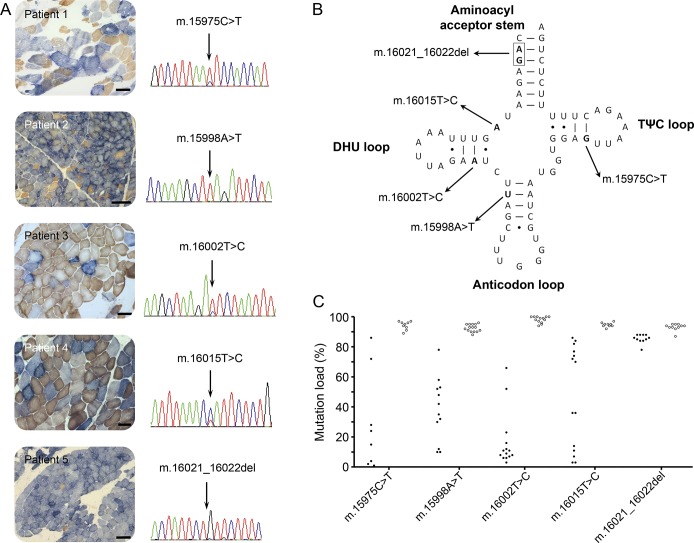
Histopathologic and molecular genetic characterization of 5 pathogenic *MT-TP* (mt-tRNA^Pro^) mutations (A) Sequential COX-succinate dehydrogenase histochemistry demonstrating a mosaic pattern of COX deficiency in all patient muscle samples (COX-deficient fibers are blue, COX-positive fibers are brown); note the presence of COX-deficient ragged-red fibers in each of the 5 biopsies (scale bar = 100 μm). Sequence electropherograms showing the relevant *MT-TP* mutation for each patient are also included. (B) Schematic representation of the cloverleaf structure of the mt-tRNA^Pro^ molecule and the corresponding location of the 5 pathogenic mutations. Each mutation occurs in a different stem of the mt-tRNA^Pro^ molecule. The affected position and the substitution that occurs are highlighted in bold. (C) Single muscle fiber mutation load segregation. The graph shows the mutation load measured in individual COX-positive (closed circles) and COX-deficient fibers (open circles) laser-microdissected from muscle biopsies of the 5 patients. In each case, the candidate *MT-TP* mutation segregates with the biochemical (COX) defect in single muscle fibers.

We identified 5 adult patients with mitochondrial disease due to mutations in the *MT-TP* gene; their clinical, histochemical, and genetic data are summarized in table e-1. Histopathologic analysis of muscle biopsies from all 5 patients revealed marked mitochondrial pathology in the form of a mosaic pattern of COX deficiency (figure 1A), which ranged from ∼16% COX-deficient fibers in patient 3 up to >80% COX-deficient fibers from patients 2 and 5. COX-deficient ragged-red fibers were noted in all patients to varying degrees, although strongly succinate dehydrogenase–reactive blood vessels were not reported (figure 1A). Quantitative fluorescence-based immunohistochemistry confirmed decreased levels of both complex I (*NDUFB8*) and complex IV (*COX-I*) subunits, in accordance with an underlying disorder of mitochondrial translation (figure e-1).

Sequencing of the entire mitochondrial genome identified candidate *MT-TP* gene mutations at evolutionarily conserved positions in all 5 patients (figure 1, A and B; figure e-2). The m.15975T>C mutation^5^ and m.16002T>C mutation^6^ in patients 1 and 3, respectively, were previously reported, while those in patient 2 (m.15998A>T), patient 4 (m.16015T>C), and patient 5 (m.16021_16022del) were novel. In all cases, the highest mutation load was detectable in muscle, varying from 25% in patient 3 to >95% in patients 2 and 5 (table e-1). Screening of additional tissues was also performed; patients 1, 2, and 3 showed restricted expression of the *MT-TP* mutation to muscle, while patients 4 and 5 showed a hierarchical mutation segregation pattern (table e-1). Single muscle fiber segregation studies demonstrated statistically significant higher mutation levels in COX-deficient fibers compared with COX-positive fibers, thus confirming pathogenicity of each *MT-TP* mutation (figure 1C; table e-2).

## Discussion.

We report 5 adult patients with mitochondrial disease due to different mutations in the *MT-TP* gene with a predominantly myopathic phenotype. Ptosis (+/− progressive external ophthalmoplegia), proximal myopathy, and marked perceived fatigue appear to be salient features. In each case, the marked degree of COX deficiency and downregulation of both complex I and complex IV subunits in muscle was strongly suggestive of a defect in mitochondrial translation and entirely in keeping with an mt-tRNA mutation. To date, only 5 patients have been reported, each with different *MT-TP* mutations, and variable clinical features have been observed including ataxia, deafness, dilated cardiomyopathy, myoclonic epilepsy with ragged-red fibers–like disease, and retinitis pigmentosa; a myopathic phenotype is reported in all cases (table e-1).

Patients 1, 2, and 3 showed restricted expression of their *MT-TP* mutations to muscle, strongly indicative of a de novo mutational event,^7^ whereas patients 4 and 5 showed a hierarchical segregation pattern as observed in many pathogenic mtDNA mutations. Screening of maternal samples was undertaken for patients 2, 4, and 5, with maternal inheritance being confirmed in patient 4 only. Of note, only one of the previous studies demonstrated maternal inheritance of the *MT-TP* mutation, with 4 of the remaining studies also reporting apparent or likely de novo mutational events.

Single muscle fiber segregation studies remain the gold standard test to confidently establish pathogenicity of novel mtDNA variants. Although the m.15975T>C^5^ and m.16002T>C^6^ mutations have been reported previously, functional studies were not undertaken to confirm pathogenicity. Subsequent studies in muscle biopsies of all 5 patients confirm that mutation loads segregated with the mitochondrial histochemical defects in muscle (figure 1C; table e-2), powerfully illustrating an ongoing requirement to access pathologically relevant tissue—skeletal muscle—to support the investigation and diagnosis of patients with mitochondrial myopathy, even in the current era of high-throughput next-generation sequencing technologies.

## Supplementary Material

Data Supplement
