# Supplementary material for: Pathogenic mtDNA mutations causing mitochondrial myopathy: The need for muscle biopsy
Source: Neurol Genet. 2016 Jun 23;2(4):e82. doi: 10.1212/NXG.0000000000000082 (PMC4972142; doi:10.1212/NXG.0000000000000082)
Supplement: Data Supplement [file supp_2_4_e82__index.html]

Data Supplement 

# Pathogenic mtDNA mutations causing mitochondrial myopathy: The need for muscle biopsy

## Data Supplement

**Files in this Data Supplement:**

- Figure e-1 - PDF
- Figure e-2 - PDF
- Table e-1 - Microsoft Word file
- Table e-2 - Microsoft Word file
