# Supplementary material for: Pathogenic mtDNA mutations causing mitochondrial myopathy: The need for muscle biopsy
Source: Neurol Genet. 2016 Jun 23;2(4):e82. doi: 10.1212/NXG.0000000000000082 (PMC4972142; doi:10.1212/NXG.0000000000000082)
Supplement: Data Supplement [file supp_2.4.e82_Hardy_et_al_Figure_e-1.pdf]

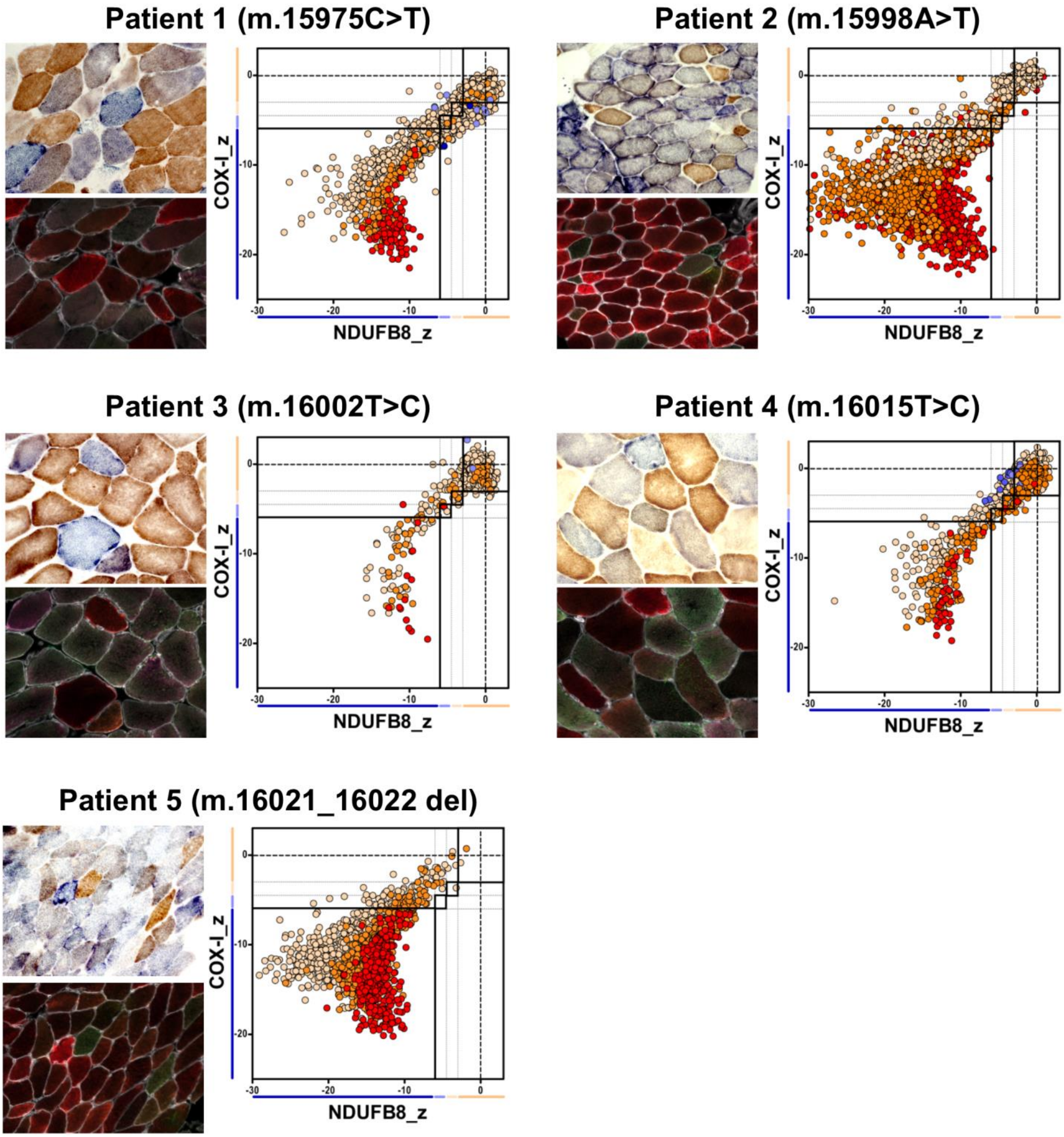

Figure e-1: Mitochondrial respiratory chain (MRC) profile from patients with novel *MT-TP* mutations confirming loss of complex I and complex IV subunits. Shown for each patient is COX/SDH histochemistry (upper panel) and quadruple immunofluorescence (lower panel: laminin (405nm) white; COX-I (488nm) green, porin (546nm) red; NDUFB8 (647nm) purple), performed in serial muscle sections obtained from each case. MRC graphs link the immunodetectable levels of COX-I, NDUFB8 and porin in patients biopsies. Each dot represents an individual muscle fibre, colour coded according to its mitochondrial mass (very low: blue, low: light blue, normal: light orange, high: orange and very high: red). Thin black dashed lines indicate the SD limits for the classification of fibres, lines next to x and y axis indicate the levels of NDUFB8 and COX-I respectively (beige: normal, light beige: intermediate(+), light blue: intermediate(-) and blue: deficient). Bold dashed lines indicate the mean expression level of normal fibres (see Rocha MC et al. Sci. Rep. 2015;5:15037 (doi: 10.1038/srep15037).
