# Supplementary material for: Pathogenic mtDNA mutations causing mitochondrial myopathy: The need for muscle biopsy
Source: Neurol Genet. 2016 Jun 23;2(4):e82. doi: 10.1212/NXG.0000000000000082 (PMC4972142; doi:10.1212/NXG.0000000000000082)
Supplement: Data Supplement [file supp_2.4.e82_Hardy_et_al_Figure_e-2.pdf]

	Acc stem	T stem	T loop	T stem	V region	Ac stem	Ac loop	Ac stem		D stem	D loop	D stem		Acc stem
**Patient 1**	TCAGAGAA	AAAGT	CTTTA	ACTCC	ACCA	TTAGC	ACCCAAA	GCTAA	G	ATTC	TAATTT	AAAC	TA	TTCTCTG
**Patient 2**	TCAGAGAA	AAAGT	CTTTA	ACTCC	ACCA	TTAGC	ACCCAAA	GCTAA	G	ATTC	TAATTT	AAAC	TA	TTCTCTG
**Patient 3**	TCAGAGAA	AAAGT	CTTTA	ACTCC	ACCA	TTAGC	ACCCAAA	GCTAA	G	ATTC	TAATTT	AAAC	TA	TTCTCTG
**Patient 4**	TCAGAGAA	AAAGT	CTTTA	ACTCC	ACCA	TTAGC	ACCCAAA	GCTAA	G	ATTC	TAATTT	AAAC	TA	TTCTCTG
**Patient 5**	TCAGAGAA	AAAGT	CTTTA	ACTCC	ACCA	TTAGC	ACCCAAA	GCTAA	G	ATTC	TAATTT	AAAC	TA	TTCTCTG
H. sapiens	TCAGAGAA	AAAGT	CTTTA	ACTCC	ACCA	TTAGC	ACCCAAA	GCTAA	G	ATTC	TAATTT	AAAC	TA	TTCTCTG
P. troglodytes	TCAGAGAA	AAAGT	AATTA	ACTTC	ACCA	TCAGC	ACCCAAA	GCTAA	G	ATTC	TAATTT	AAAC	TA	TTCTCTG
P. paniscu	TCAGAGAA	AAAGT	CTTTA	ACTCC	ACCA	TCAGC	ACCCAAA	GCTAA	G	ATTC	TAATTT	AAAC	TA	TTCTCTG
H. lar	TCAGAGAA	AAAGT	ACTTA	ACTTC	ACCC	TCAGC	ACCCAAA	GCTAA	A	ATTC	TAACTT	AAAC	TA	TTCTCTG
M. musculus	TCAAGAAG	AAGGA	GCTAC	TCCCC	ACCA	CCAGC	ACCCAAA	GCTGG	T	ATTC	TAATT	AAAC	TA	CTTCTTG
R. norvegicus	TCAAGAAG	AAGGA	ACTACC	TCCCC	ACCA	TCAAC	ACCCAAA	GCTGA	T	ATTC	TATTT	AAAC	TA	CTTCTTG
B. taurus	TCAAGGAA	GAAAC	TGCA	GTCTC	ACCA	TCAAC	CCCCAAA	GCTGA	A	GTTC	TATTT	AAAC	TA	TTCCCTG
G. gallus	TCAGAAAA	GGAGG	GCTCAAA	CCTCC	ATCT	CCAGC	TCCCAAA	GCTGG	T	ATTT	TCAAAT	AAAC	TA	CTCTCTG
G. morhua	TCAGAGGG	AAGAG	ATTTTAA	CTCCC	ACCA	CTAGC	TCCCAAA	GCTAG	A	ATTC	TAAATT	AAAC	TA	CCCTCTG
D. melanogaster	TCAAGAGA	AAAGA	AATT	TCTTT	TTCA	TTAAT	CCCCAAA	ATTAA	T	ATTT	TAAAT	AAAC	TA	CCTCTTG

Figure e-2. Evolutionary conservation of the *MT-TP* (mt-tRNA^{Pro}) gene illustrating the location of the five pathogenic mutations identified in this study. Phylogenetic analysis of the *MT-TP* gene reveals that the positions of each mutation (highlighted in red) are highly-conserved, providing further support for pathogenicity.
