# Supplementary material for: Pathogenic mtDNA mutations causing mitochondrial myopathy: The need for muscle biopsy
Source: Neurol Genet. 2016 Jun 23;2(4):e82. doi: 10.1212/NXG.0000000000000082 (PMC4972142; doi:10.1212/NXG.0000000000000082)
Supplement: Data Supplement [file supp_2.4.e82_Hardy_et_al_Table_e-1.docx]

| **Patient Details^§^ (Sex/age of onset (y))** | **Clinical Presentation^*^** | **Family History** | **Mutation** | **Previously published?** | **Muscle Biopsy findings^#^** | **Mutation Load (%)** | | **Inheritance Pattern^** |
| --- | --- | --- | --- | --- | --- | --- | --- | --- |
|  |  |  |  |  |  | **Patient Tissues^†^** | **Familial Tissues^**^** |  |
| 1 (F, 20s) | Isolated PEO, ptosis | No | m.15975C>T | Da Pozzo *et al.* (2009) | >50% COX-deficient fibres, 12% RRF | M: 79%  B: 0% | Seven unaffected siblings – not tested | Unknown, likely sporadic |
| 2 (M, Teens) | Mild asymmetric proximal myopathy | No | m.15998A>T | Novel | >80% COX-deficient fibres and RRF;  complex I+III+IV defect | M:95%  U: 0%  BM: 0%  B: 0% | Unaffected mother –  B: 0%; U: 0%; BM: 0% | Sporadic |
| 3 (F, mid 30s) | PEO and asymmetric ptosis; proximal myopathy; absent LL reflexes; mild oropharyngeal dysphagia; respiratory muscle weakness; prominent fatigue. | Not known | m.16002T>C | Seneca *et al.* (2000) | 16% COX-deficient fibres , 4% RRF | M: 25%  U: 0%  BM: 0%  B: 0% | Not tested | Unknown, likely sporadic |
| 4 (M, N/A) | PEO, proximal myopathy, exercise intolerance | No | m.16015T>C | Novel | 25% COX-deficient fibres, 5% RRF | M: 70%  U: 37%  BM: 15%  B: 8% | Unaffected mother –  U: 4%; BM: 4%; B: 0% | Maternal |
| 5 (F, 14y) | Mild retinitis pigmentosa, SNHL; seizures; mild ptosis, small stature, exercise intolerance/fatigue | No | m.16021_16022del | Novel | 98% COX-deficient fibres, 10% RRF; complex I+IV defect | M: 95%  U: 56%  BM: 35%  B: 24%  H: 9% | Unaffected mother –  U: 0%; BM: 0%; B: 0%; H: 0% | Sporadic |
| 6 (F, 27) | MERRF-like disease, myoclonic jerks, seizures, cerebellar ataxia, bilateral SNHL, dysarthria, proximal muscle weakness | No | m.15967G>A | Blakely *et al.* (2009) | >10% COX-deficient fibres; complex I defect | M: 69%  U: 10%  B: 0% | Unaffected mother –  U: 0%; B: 0% | Sporadic |
| 7 (F, 40) | Severe muscle weakness, ataxia, retinitis pigmentosa, bilateral hypoacusia, bilateral SNHL, bilateral nystagmus, dysarthria | No | m.15975C>T | Da Pozzo *et al.* (2009) | 5% COX-deficient fibres, 2% RRF;  complex I defect | M: 40%  U: 40%  BM: 30%  B: <10%  H: 0%  F: 0% | Patient’s unaffected sister and daughter –  U: 0%; BM: 0%; B: 0%; H: 0% | Unknown |
| 8 (F, 7) | Pure myopathy; no ophthalmoparesis | No | m.15990G>A | Moraes *et al.* (1993) and  Ionasescu *et al.* (1994) | COX-deficient and RRF fibres (not quantified);  complex I+III+IV defect | M: 85%  B: 0% | Unaffected mother –  B: 0% | Sporadic |
| 9 (M, 3) | Fatigue/weakness, exercise intolerance, muscle weakness, bilateral ptosis, vertical ophthalmoplegia | No | m.16002T>C | Seneca *et al.* (2000) | RRF fibres (not quantified) | M: 70%  B: 0% | Unaffected mother –  B: 0% | Sporadic |
| 10 (F, 35) | Proximal muscle weakness, migraine, pigmentary retinopathy, deafness, leukariosis on MRI | No | m.16023G>A | Blakely *et al.* (2013) | 65% COX-deficient fibres, 5% RRF | M: 86%  U: 36%  B: 9% | Unaffected mother –  U: 7%; B: 0% | Maternal |

***Table e-1: Genotype and phenotype data from the five patients included in this study (1–5) and the five previously-reported patients (6–10) harbouring pathogenic MT-TP mutations.*** Patient details (**§**) include ID number, sex, and age at first presentation; a variety of clinical presentations were seen across this case series (*****), PEO = Progressive External Ophthalmoplegia, SNHL = Sensorineural Hearing Loss. The percentage of COX-deficient fibres and ragged-red fibres (RRFs) in the muscle biopsy were determined, whilst respiratory chain enzyme data were available for 2 patients (P2 and P5) confirming a generalised disorder of mitochondrial translation (**#**) and also confirmed by immunohistochemical assessment. Mutation loads were determined in a variety of tissues from the patient (**†**) and maternally-related individuals (******). Tissues examined included skeletal muscle (M), blood (B), urine (U), buccal mucosa (BM), hair shafts (H) and fibroblasts (F). The pattern of inheritance within the family for each mutation was determined where possible based upon mutation loads in familial tissues (**^**).

**References**

1. Da Pozzo P, Cardaiolo E, Malfatti E, et al. A novel mutation in the mitochondrial tRNA(Pro) gene associated with late-onset ataxia, retinitis pigmentosa, deafness, leukoencephalopathy and complex I deficiency. Eur J Hum Genet 2009;17(8);1092-1096

2. Seneca S, Ceuterik-De Groote C, Van Coster R, De Meirleir, L. A novel mitochondrial transfer RNA proline mutation. J Inherit Metab Dis 2000;23(8);853-854

3. Blakely EL, Trip SA, Swalwell H, et al. A new mitochondrial transfer RNAPro gene mutation associated with myoclonic epilepsy with ragged-red fibers and other neurological features. Arch Neurol 2009;66(3);399-402

4. Moraes CT, Ciacci F, Bonilla E, Ionasescu V, Schon EA, DiMauro S. A mitochondrial tRNA anticodon swap associated with a muscle disease. Nat Genet 1993;4(3);284-288

5. Ionasescu VV, Hart M, DiMauro S, Moraes CT. Clinical and morphologic features of a myopathy associated with a point mutation in the mitochondrial tRNA^Pro^ gene. Neurology 1994;44(5):975-977.

5. Blakely EL, Yarham JW, Alston CL, et al. Pathogenic mitochondrial tRNA point mutations: nine novel mutations affirm their importance as a cause of mitochondrial disease. Hum Mutat 2013;34(9);1260-1268
