# Supplementary material for: Pathogenic mtDNA mutations causing mitochondrial myopathy: The need for muscle biopsy
Source: Neurol Genet. 2016 Jun 23;2(4):e82. doi: 10.1212/NXG.0000000000000082 (PMC4972142; doi:10.1212/NXG.0000000000000082)
Supplement: Data Supplement [file supp_2.4.e82_Hardy_et_al_Table_e-2.docx]

| **Patient** | **Mutation** | **Percentage Mutation Load (%)** | | **P-value** |
| --- | --- | --- | --- | --- |
|  |  | **COX-positive fibers** | **COX-deficient fibers** |  |
| Patient 1 | m.15975C>T | 25.8 ± 10.7 (n=9) | 94.2 ± 0.9 (n=9) | p<0.0001 |
| Patient 2 | m.15998A>T | 38.33 ± 6.09 (n=12) | 92.47 ± 0.62 (n=15) | p<0.0001 |
| Patient 3 | m.16002T>C | 17.0 ± 4.99 (n=14) | 98.15 ± 0.58 (n=13) | p<0.0001 |
| Patient 4 | m.16015T>C | 46.8 ± 9.1 (n=14) | 94.7 ± 0.5 (n=9) | p=0.0004 |
| Patient 5 | m.16021_16022del | 85.5 ± 0.7 (n=11) | 93.2 ± 0.9 (n=11) | p<0.0001 |

**Table e-2.** Summary of single muscle fiber mutation load data. The mutation load in individual COX-positive and COX-deficient fibers is shown for each of the five pathogenic mutations identified in this study (± standard deviation). Each mutation showed statistically-significant segregation with the biochemical defect in muscle at the 95% confidence level.
